# Some Experimental Tests with Sterilizing Agents

**Published:** 1895-01

**Authors:** L. P. Bethel

**Affiliations:** Kent, Ohio


### Some Experimental Tests with Sterilizing Agents.

BY L. P. BETHEL, D.D.S., M.D., KENT, OHIO.

Read before the Ohio State Dental Society, December, 1894.

The object of these experiments has been to find, if possible, some chemical agent that, for the sterilization of dental instruments, would prove as efficient as boiling water.

A chemical disinfectant always at hand and ready for use would be, in many ways, an advantage.

The agents used in these tests, were electrozone, formalin, kerosene oil and peroxid of sodium.

The latter was used in two ways: As a saturated solution, made in the manner described by Dr. Van Woert, and by putting a small quantity, about as much as can be placed on a silver dime, into a glass containing infected instruments and filled about one-third full with water. The gases, liberated by the chemical action, acting in their nascent state on the infectious material.

### METHOD OF PROCEDURE.

In order to have the tests conform as nearly as possible to actual practice, a number of decayed teeth were taken immediately after extraction and placed in a test tube containing sterilized meat boullion.

The teeth used presented various stages of decay and suppuration of the pulps. Previous to inserting the teeth in the boullion, however, the decay was well loosened, by means of a sterilized excavator, to insure a distribution of many bacteria in the solution. The contents of the test tube were well mixed and trial cultures made on gelatin and boullion to determine whether the bacteria, in the solution, would grow on these media.

The result was that the germs developed on gelatin, liquefying it quite rapidly at room temperature, but thrived better in boullion kept a little below blood temperature in an incubator.

The instruments used for the experiments were a barbed broach, a large bur, and an excavator. These were dipped in the tooth mixture, allowed to dry in air and then immersed in the disinfecting fluid. A stab culture was then made in gelatin tube No. 1, the instrument carried to tube No. 2, and both smear and stab cultures made, then carried to still another tube and this repeated.

Three tubes were used for the reason that enough of the disinfectant might be carried into tube No. 1 to prevent the growth of germs, should any remain alive, as most of the sterilizing agent would remain in this tube after a stab culture had been made, and yet there would still remain some germs on the instrument to be carried to one or both of the other tubes, yet not enough of the disinfectant to prevent growth.

Where boullion was used two tubes only were employed, for the disinfectant would be rinsed off in tube No. 1 and any remaining germs carried to tube No. 2 would not be prevented from multiplying. It is probable that the small amount of disinfecting fluid carried into tube No. 1 would become so diluted with the boullion that it would have no further action than to somewhat retard the growth, yet, to be certain, a second tube was used.

The time of immersion in the disinfectants was three minutes, except in a few instances, for, to meet the demands of the dentist, an agent should completely sterilize an instrument in a few minutes at the most.

Aside from tooth bacteria the *subtilis bacilli*, containing very resistant spores, and the *staphylococcus pyogenes*, were used in some of the experiments. Both of these bacteria grow rapidly and being so resistant further tested the value of these chemical agents as sterilizers.

The results of the experiments were as follows:

| DISINFECTANT. | CULTURE MEDIA. | CULTURE. | Time of exposure, minutes. | Number of Cultures. | Sterile. | Non-sterile. |
|---|---|---|---|---|---|---|
| Formalin | Gelatin | Tooth bacteria | 3 | 3 | 3 | |
| do | Boullion | do | 3 | 6 | 6 | |
| do | do | Subtilis | 3 | 2 | 2 | |
| do | Gelatin | Staph. pyogenes | 3 | 6 | 4 | 2 |
| Formal'n (washing infected inst. before sterilizing.) | do | Suppurating pulp | 3 | 2 | 2 | |
| Electrozone | Gelatin | Tooth bacteria | 3 | 3 | 3 | |
| do | Boullion | do | 3 | 6 | 4 | 2 |
| do | do | do | 1 | 1 | | 1 |
| do | Gelatin | Subtilis | 3 | 3 | 3 | |
| do | Boullion | do | 3 | 4 | 4 | |
| do | Gelatin | Staph. pyogenes | 3 | 5 | 3 | 2 |
| do | do | do | 5 | 3 | 3 | |
| do | Boullion | do | 3 | 2 | 2 | |
| Electrozone (washing infected inst. before sterilizing.) | Gelatin | Suppurating pulp | 3 | 2 | 2 | |
| Sodium peroxid (saturated solution.) | Gelatin | Tooth bacteria | 3 | 6 | 6 | |
| do | Boullion | do | 3 | 2 | | 2 |
| do | Gelatin | Staph. pyogenes | 3 | 6 | 6 | |
| do | Boullion | do | 3 | 2 | | 2 |
| Sodium peroxide (saturated solution—washing infected inst. before sterilizing.) | Gelatin | Suppurating pulp | 3 | 2 | 2 | |
| Sodium peroxid placed in water containing inst. | Boullion | Tooth bacteria | 3 | 2 | | 2 |
| do | Gelatin | Staph. pyogenes | 3 | 2 | 2 | |
| Kerosene | Gelatin | Tooth bacteria | 3 | 6 | | 6 |
| Kerosene (washing infected inst. before sterilizing.) | do | Suppurating pulp | 3 | 2 | 2 | |

It will be observed that in the use of kerosene in six cultures all were non-sterile, while in two other tests with this agent, precaution being taken to wash the infected instrument in hot water before immersing in kerosene, both cultures remained sterile.

This suggests the advantage derived from the thorough washing of infected instruments prior to immersing in the disinfecting fluid, for it takes away the accumulated mass of infectious material so that those bacteria remaining are more separated and therefore more readily acted upon by the sterilizing agent.

In two of the tests with formalin, and two with electrozone where staphylococcus pyogenes was used, the non-sterility was due, doubtless, to having taken the bacteria directly from potato culture where the growth was abundant and the amount taken so massed that the disinfectants could not penetrate to all the germs in the short time of exposure allowed.

From these tests it will be seen that formalin, electrozone and sodium peroxid all have a high disinfecting power and may be employed with advantage in dentistry for other purposes than the sterilizing of instruments.

You are probably all familiar with peroxid of sodium from what Dr. E. C. Kirk and others have written about its use in dentistry as a tooth bleacher, pulp canal cleanser, etc.

The attention of the dental profession was called to the use of formalin in dentistry by Dr. J. S. Cassidy, in an article on Formaldehyd in the October issue of *The Ohio Dental Journal.* The characteristics and action of formalin are fully described in this contribution and it will bear careful perusal. Dr. Cassidy has found much satisfaction in the use of this drug in the treatment of pulpless teeth, etc.

Formalin is acid in reaction, but only a very slight coagulator of egg albumen. Its odor, while not unpleasant, is somewhat irritating to the membranes of the nose and throat.

Dr. C. F. W. Bodecker has called attention to the use of electrozone, in an article published in the October number of *The Dental Practitioner aud Advertiser.* He highly recommends it for the treatment of pulpless teeth, pyorrhœa alveolaris, as a mouth wash, etc.

It is neutral in reaction, a non-coagulator of albumen, and has marked bleaching qualities. It has a chlorin odor but instruments immersed for days in the liquid show but slight action of the drug.

Electrozone is prepared by treating sea water with electricity. It contains 3½ per cent. of chlorids, sodium, potassium, magnesium, calcium in solution, and iodin and bromin. Sea water being an unknown quantity the preparation may contain some other ingredients. It is non poisonous and may be used internally as well as externally. When not in use it should be kept well corked and in a cool place. Its efficacy as a disinfectant, its wide range of use and cheapness of the preparation, 50 cents per quart bottle, make it a valuable addition to the list of useful dental medicines.

DISCUSSION.

Dr. H. A. Smith, Cincinnati: I think it must be very gratifying to all of us to have a paper like this presented to a body such as we are members of. Knowledge first hand I think is very scarce with us. I feel like bowing very low indeed to a man like this who will make original investigations in this direction, that are interesting and exceedingly valuable. The one thing that concerns us most in the practice of dentistry to-day is the need of an efficient, rapid method of sterilization. Do we ever think how difficult it is to produce cleanliness? It is almost impossible to obtain surgical cleanliness. When we have washed our hands thoroughly and applied the rubber dam, in the act the fingers have come in contact with parts of the mouth, and they are no longer clean. The moment you take up the sterilized cotton with the fingers that have come in contact with the mouth, you have infected the cotton, and putting it in the tooth you infect that tooth. There is nothing about the dental office perhaps, that has not organisms.

Only as we are clean do we succeed as dentists. The crying need is a sterilizing agent that will sterilize instruments quickly. The dentist is a busy practitioner and can't afford to wait for anything. Three minutes is a long enough time and it should not exceed ten minutes to produce perfect sterilization. I don't mean the cleanliness that stands next to Godliness. We have some experiments in this direction, notably of Dr. Miller where he has formulated a method for us. I believe he experimented some time in sterilizing remains of devitalized pulps in root canals. In treating the remains of pulp tissue with a disinfectant we make a filling and it would take six months or a year to know whether it was a success or not. I would not like to make such experiments without knowing whether it was going to be successful.

Here we have something tangible. Think of the cases that could be treated for the poor if we had an agent of this sort. Nothing consumes so much time as the treatment of root canals. Don't you feel like bowing low to a man who will make these experiments for us—original research.

I am not competent to discuss the paper thoroughly. I think those three agents he discusses command our attention, and we should test them, making a note of each case we try.

Dr. J. R. Callahan, Cincinnati: I have been on the floor so often that I am ashamed of myself. I am greatly pleased with the paper and also obliged for the work Dr. Bethel has done, and when the paper appears in print I shall study it closely.

Dr. C. R. Butler, Cleveland: I am something like Dr. Smith; I certainly will bow to the compliment that has been paid us here this morning by Dr. Bethel. He has gone into this subject to a considerable extent, and it is of an original character, and we congratulate ourselves as a society on such work done by one of its members. I could say much in a general way, but it has been presented in such a manner that we will all be profited by it. The paper ought to be published largely in the State, and we ought to be able to apply it in every-day practice, for when the sterilization of our instruments can be done so rapidly, the necessity of it we can see more and more.

Dr. W. B. Ames, Chicago, asked Dr. Bethel the degree of heat that is required to thoroughly sterilize instruments.

Dr. L. P. Bethel, Kent: That depends largely upon the bacteria with which the instrument is infected. There are some bacteria that are very resistant and some that are not so much so. In the mouth we find almost everything at times, though perhaps not constantly. The bacteria are taken in with the breath from the air, dust, etc. In that way we find many kinds of bacteria in the mouth, not normally there, and it is hard to tell the exact degree of heat that would destroy all of them, but a temperature 300° to 500° F. would probably do so.

Dr. Ames: Do you not find that disinfecting agents are more potent when they are warm?

Dr. Bethel: Yes sir.

Dr. W. B. Ames, Chicago: I have thought a little and experimented on the subject, but not in the way Dr. Bethel has. I have a sterilizing agent that I have employed to some extent and that is glycerine heated to about 300° F. An apparatus can be made by taking a porcelain dish of some sort and placing in it the glycerine and into this a tall glass beaker containing water. As the glycerine is heated to the point that will cause water to boil, the glycerine will be found to be very much hotter, will have almost reached the boiling point, yet will have no disagreeable odor, as the boiling water keeps down the temperature of the glycerine. I have immersed the instruments in this glycerine, and about 300° will thoroughly sterilize them.

I have done considerable sterilizing in root canals of putrescent pulp tissue which has been only partially removed, it being impossible to thoroughly remove all. The fact of iodine being as potent as it is, it being a germicide and undisputed deodorant, the efficacy of oil of cassia and the fact of iodine being soluble in oil of cassia, led me to make my experiments with them, and I find that the solution of iodine and oil of cassia seems to have certain peculiarities. The hydro-carbon combinations give various compounds, and I believe from what I have seen from making a solution of iodine in oil of cassia a new compound is formed to which your attention has never been called. You can dissolve two grains of iodine in a dram of oil of cassia and get a syrupy solution. Use more than two grains of iodine and in a few days you will have a hard mass. If you use five grains or ten grains you will have a mass like so much anthracite coal, insoluble, and that fact leads me to believe a thoroughly new compound has been formed. From six or eight minutes use of this, I have obtained results in the disinfection of canals that would lead me to think it is the most efficient combination I have used for that purpose. It is very convenient to use, as you can use any kind of point. I want to speak of its being soluble and yet so slightly soluble in water that we can place it in the root and leave it for a long time without being dissolved.

Dr. C. R. Butler, Cleveland: I am glad Dr. Ames has spoken of his experiment. A certain amount of iodine to a dram gives this undesirable mixture. I have been using it and experimenting with it and flattered myself it was doing good work, as Dr. Ames has said, and he has been working in the same direction. I am not particular about the small quantity of the iodine, but I don't carry it to the soluble point.

Dr. Henry Barnes, Cleveland: I have found the same results from the use of iodine and oil of cassia. It resulted in this solid mass, but I have not found that it deteriorated in that condition. I have used it in children's teeth.

So far as Dr. Bethel's paper is concerned, I want to voice what the others say in regard to it. If there was no other paper before this society, if we could go home and take the thoughts brought out in this, and use them, it would more than pay us for the trouble and time expended here.

Dr. F. E. Battershell: I was very much pleased with the Doctor's paper. I suppose none of us could have an adequate idea of the pains-taking and care of these researches. I think that these contributions are of great benefit. We cannot all of us experiment in every line, and when they are laid before us in that way they are very valuable.

We sterilize the point or ball of an instrument and then we handle the instrument and transfer our hands from the mouth to the instrument, which in handling becomes more or less contaminated, but we only disinfect a portion of this. By making a box in which we could lay our instruments and having them constantly at a heat that will sterilize, we will completely obliterate all germs that are deposited on instruments and then we have an ideal sterilizer. Wouldn't it be well to lay these things in the sweat boxes and our clothing, or coat, because we are not only responsible for what we put into the mouth but we are responsible for what we place in close contact with patients. If we have been operating upon a patient who may have disease germs about the clothing and we transfer ourselves to another person who, being very susceptible to the disease, may become infected by a transportation of the disease germs, we can avoid the infection by taking the outer robe or coat and placing it in a sweat box and sterilizing it before going to the other patient. Wouldn't it be a good thing?

Dr. ———: Wouldn't it be a good idea to put the patient in that sweat box?

Dr. G. E. Hunt, Indianapolis: I consider this one of the most valuable papers on the subject that has ever been presented.

The experiments show the greatest care and the results arrived at are very important. The subject of sterilizing our instruments should receive a great deal of attention. The results that Dr. Bethel has reached are results that should be retained in the practice of all of us. There is no doubt heat is the best sterilizing instrument we have. If we heat anything hot enough it will kill all the bacteria in it. It will be impracticable to heat everything, and many bacteria even half an hour's steaming will not kill. We don't want to spare the instrument for half an hour or twenty minutes even. In heating, it would require such cumbersome apparatus as to be impracticable, and if we could avoid it we would be better off. The ideal sterilizer will be something in the line the Doctor has been experimenting in that will thoroughly sterilize in a short time.

Electrozone promises great things. I think it was first used to sterilize a large mass of putrid matter in Ricker's Island Extension, East River, N. Y. The place had been used for a dumping ground for all Manhattan. After a long time it was discovered that the hot sun made it a mass of corruption and the wind blew it over New York City. Somebody conceived the idea of making electrozone out of sea water and then sterilizing this putrid mass with the electrozone.

From the experiments that have been made I would judge that formalin was also a formidable drug.

I think that this matter of sterilizing can be carried further than is necessary. The gentleman who preceded me spoke about sterilizing our coats. I don't thing that is necessary. We may come to it after a while, We are in danger of carrying certain lines of practice to excess. To sterilize the napkin after it comes from the wash may be necessary, but I don't believe it. I believe a napkin, after it has been washed, is sterilized enough for common use. It is different from instruments. They are put into the human mouth and they require sterilizing.

Dr. J. Taft, Cincinnati: As heat is conceded by all to be the best sterilizing agent, the only question being to make it practicable, it seems to me that Dr. Custer might make some arrangement for sterilizing by electrical heat that will be practicable and better, I think, than anything else.

Dr. C. H. Harroun, Toledo: I would like to suggest in regard to the napkins, if Dr. Hunt would go into a laundry and notice the way in which our napkins are washed, he would think it was necessary to sterilize them. The Chinaman takes a pail of water, takes some in his mouth and squirts it out on the napkins before ironing, and then what have you got?

Dr. Hunt: I have my washing done at home and I know what I get.

Dr. Bethel: I appreciate the remarks that have been made and thank the speakers for them. In regard to these experiments I wish to say a few words. They were made under the most unfavorable circumstances. The instruments were infected and the infectious material was left on them; they were not washed before putting in the sterilizing fluid. The cultures were placed in bouillon instead of sterilized water. When placed in sterilized water the growth is hindered and the bacteria are less resistant. For example, take anthrax bacilli and the spores are so much more resistant in bouillon than sterilized water that it requires twenty times as much bichloride of mercury to destroy them as when used in sterilized water.

This was done to possibly offset the resistance of the bacteria as they are found in their natural habitat, and where they feed upon natural food. They are more resistant there than when cultivated on any artificial media. I have little doubt but that had the instruments been thoroughly washed we would have found them to have been sterilized with three minutes exposure.

I am glad to hear of Dr. Ames' solution. The only objection I would raise to the use of oil as a sterilizing agent would be that the instrument or surface should be thoroughly dried before using the disinfectant fluid, because oil and water are incompatible. If there was much moisture present it would not permit it to be as effectually acted upon, as it would if the water was gotten rid of.

In regard to the electrical apparatus, it would undoubtedly be one of the finest, but unfortunately all dentists do not have the advantages of the electric current, particularly in the small towns, and it would not be practical there, unless however, it could be used with a storage battery or something of that sort. Yet these are, in themselves, hard to keep in order.

In regard to wiping the instruments after sterilizing, I have been in the habit of using cottonoid instead of napkins. I think there can be no germs in cottonoid to do any harm, as it must be thoroughly sterilized when made, and then there would be no nourishment left in the cotton, to make the growth effectual, because all the oils and fats have been taken out of it. Perhaps a better way to dry instruments would be by hot air.

